# Supplementary material for: Using virtual reality for anatomical landmark annotation in geometric morphometrics
Source: PeerJ. 2022 Feb 7;10:e12869. doi: 10.7717/peerj.12869 (PMC8830334; doi:10.7717/peerj.12869)
Supplement: Supplemental Information 1 — Design and implementation of the virtual reality platform used for placing landmarks, and its comparison to the desktop software Stratovan Checkpoint and a 3D digitizer arm. [file peerj-10-12869-s001.pdf]

# Virtual reality annotation system

**Dolores Messer[1,*], Michael Atchapero[2,*], Mark Bo Jensen[1], Michelle Strecker Svendsen[3], Anders Galatius[4], Morten Tange Olsen[3], Jeppe Revall Frisvad[1], Vedrana Andersen Dahl[1], Knut Conradsen[1], Anders Bjorholm Dahl[1], and Jakob Andreas Bærentzen[1]**

[1]**Technical University of Denmark, Department of Applied Mathematics and Computer Science, Kongens Lyngby, Denmark**
[2]**University of Copenhagen, Department of Psychology, Copenhagen, Denmark**
[3]**University of Copenhagen, Globe Institute, Copenhagen, Denmark**
[4]**Aarhus University, Department of Ecoscience, Roskilde, Denmark**
[*]Both authors contributed equally to this research.

Supplement to 'Virtual reality in geometric morphometrics'.

## INTRODUCTION

Using a VR platform for annotation of landmarks has several benefits. Firstly, the physical positions and rotations of the headset and the controllers can be tracked. Secondly, users get a closer-to-real-world experience in the virtual environment, which contains the item to be annotated. We present the design and implementation of a VR system with a grey seal skull as an example of the item to be annotated.

## DESIGN

In the virtual environment, the user has two virtual hands that can grab the digital skull in order to translate and rotate it. Due to the six degress of freedom (DOF) of each controller, one hand can manipulate the skull, while the other hand is used for annotation. We add another DOF so that the user can uniformly scale the skull by using two buttons on the left hand controller. Enlarging the skull lets the user locate landmark points more easily, while shrinking the skull gives an overview and improves navigation. The thumbsticks on the two Touch Controllers can also manipulate the skull by translation (left hand) and rotation (right hand).

For annotation, one hand is equipped with a ray-gun, which is a Touch Controller equipped with a laser-tip. The ray-gun casts a red ray from its tip and this can intersect with the surface of the skull. To place an annotation point, the user aims by pointing the controller at the landmark location, and presses the index trigger of the controller. For the sake of this study, given that not all operators were experienced in VR, the design was kept as simple as possible: The controller resembles a ray-gun, which links the motion of annotating a point to the act of shooting a gun. Additionally, the user can shoot already placed annotation points to move or delete them.

The design of our VR annotation software is intended to be fair in regard to the comparison to Stratovan Checkpoint, a commonly used desktop software for digitally annotating landmarks on 3D models. In our experiment, regardless of platform, we expect the operators to pick the correct skull, and use the same landmark order for all annotation rounds. Neither application guides or instructs the operators, and they must freely place landmarks on the surface of the skull. Both applications can reset the camera to a default state, and there are no other camera-control shortcuts. Finally, the control scheme cannot be reconfigurated in either application.

## IMPLEMENTATION

Our VR annotation system was developed in Unity 2019 (Unity Technologies, San Francisco, CA, USA; https://unity.com), and is targeting the Oculus Rift hardware (Facebook Technologies, LLC, Menlo Park, CA, USA; https://www.oculus.com). The Oculus Integration SDK package provided the basis for the implementation, such as tracking of the headset and controllers, as well as for the 3D models and animations for the hands and touch controllers in the virtual environment. Firstly, the grabbable object needs the hand-grabbing interaction, otherwise it should stay still. The hand-grabbing interaction

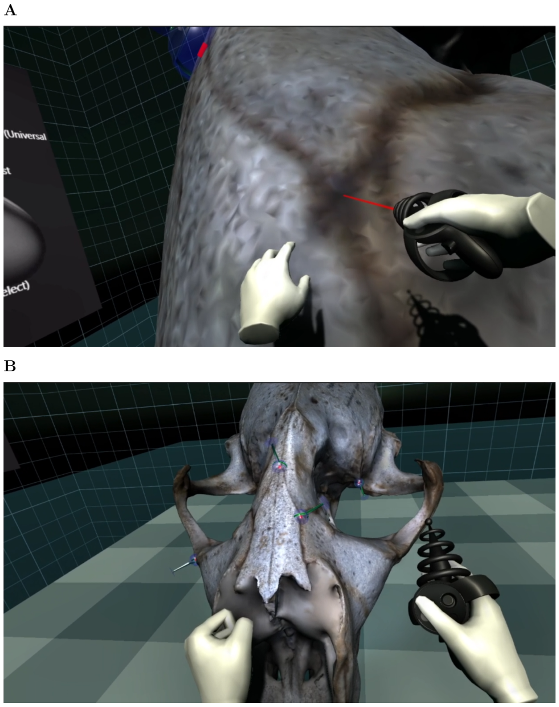


**Figure 1.** **Screenshots of the virtual environment. (A)** The user is about to place a landmark with the ray-gun. **(B)** The user has placed six landmarks on the 3D model.

requires physics (Unity's Rigidbody component), but its gravity should be disabled, and its velocities set to zero when not grabbed in every update frame. This prevents forces, velocities and interactions, such as throwing and punching the annotation object into space. Secondly, we take advantage of Unity's hierarchy and parenting system, which allows developers to order game objects in a tree structure. All objects are subject to any translations, rotations and scalations of their parent objects. For example, each annotation point on the skull is a child object of the skull, meaning that any transformation of the skull also applies to all placed annotation points.

Starting with a grabbable object using a box-collider for the hand-grabbing interaction, we added the skull as a child object with a mesh collider for annotating the surface of the skull. We experienced that the 3D models were not centred around their own origin, so we recentred and rotated the child object representing the skull appropriately to the user in the editor. We could have centred the 3D models around their origin point, however, we decided against modifying the original 3D models, because we relied on previous annotation data from Stratovan Checkpoint during the implementation. Proper use of Unity's object layers ensure that the ray-casting from the user's controller hits the mesh collider of the skull, and not the box-collider used for hand-grabbing. Finally, any placed annotation points are instantiated as child objects of the skull, with a rotation facing the surface normal of the polygon that was hit on the skull.

## COMPARISON TO DESKTOP SOFTWARE

A commonly used software for digitally annotating landmarks on 3D models using mouse and keyboard is Stratovan Checkpoint (Stratovan Corporation, Davis, CA, USA; https://www.stratovan.com/products/checkpoint). Between annotation in VR and in Stratovan Checkpoint, there are two major differences: the annotation procedure, and the view control. Table 1 provides an overview.

### Annotation procedure

To place a landmark in Stratovan Checkpoint, the user places the cursor on the surface of the skull and holds Shift while clicking. In our VR annotation software, the user aims the right hand controller at the location on the mesh surface where he or she wishes to place a landmark. As visual feedback, a red laser is intersecting the surface at this location. Annotating with a mouse on a 2D display monitor forces the

**Table 1. Summary of system core features and their differences in practice.** We note that downsampling of the 3D models was required in case of the VR annotation system, as our hardware could not render the original meshes. For consistency, we used the downsampled 3D models for data collection with both systems.

| Core Feature | Stratovan Checkpoint | Virtual Reality |
|---|---|---|
| Annotation Procedure | Mouse clicks | Ray casting from controller |
| Specimen | Downsampled 3D model used for consistency | Downsampled 3D model |
| User Interface | Overlay on-screen | In virtual environment |
| View Control | Transformation of camera | Head motion and transformation of skull |
| Translation and Rotation | Camera only | Camera and skull |
| Scaling | Camera distance only | Camera distance and skull scaling |

user to aim with the cursor from the view of the camera, which makes hitting the right surface difficult when the geometry in the area is complex. This is different in our VR annotation software, as the user can look from a different angle than where he or she is aiming from. This allows e.g. the user to look perpendicularly to the controller to verify that the red laser intersects with the correct surface. However, in both Stratovan Checkpoint and VR, the user may move around annotation points, and the annotation points will always be angled to the surface normal. Thus, the option to correct misplaced annotation points remains.

### View control

In Stratovan Checkpoint, the camera centres on an invisible point in 3D space. Holding the left mouse button and moving the mouse rotates the camera on the vertical and lateral axes around the camera's focus point. Scrolling the mouse wheel places the camera closer or farther away (dollying, not zooming, as the field of view stays the same); pressing down the mouse wheel and moving the mouse rolls the camera (rotation around the longitudinal axis). It is important to note that in Stratovan Checkpoint, looking around the skull is manipulation of the camera position and orientation, and not manipulation of the skull. In VR, the camera pose is tied to the physical location of the headset the user is wearing. However, rather than moving around to get a good angle, we instead instructed users to manipulate the position, rotation, and scale of the skull itself. Thus, view control in VR is an interplay of skull control and head motion, rather than orbiting the camera around a skull in a fixed position.

In principle, the precision in our VR prototype can be chosen arbitrarily, as the 3D model can be scaled as the user sees fit. In practice, operators enlarged the 3D model of the skull, but not arbitrarily much. The benefit of scaling the skull is that it eliminates issues with the camera's near-clipping plane. In Stratovan Checkpoint, the specimen cannot be scaled. Once the camera gets too close to the specimen, parts of the surface will be clipped and no longer visible, which makes annotation impossible. This places a cap on how closely the camera can view the object, which may pose problems for small specimens. Allowing the user to scale the annotation object, as implemented in our VR prototype, makes the application agnostic about specimen size.

Finally, programs displayed on computer monitors often display information in the user interface (UI). Stratovan Checkpoint's UI lists annotation points to track the number of annotations placed. The UI also shows the planar outlines of the skull from the point-of-view of each axis, although in this study, operators did not use it as an aid for annotation. VR applications, in general, tend to place UIs in the virtual environment rather than on-screen (Dominic and Robb, 2020). We minimized UI use to avoid breaking immersion and text possibly being difficult to read. In our VR annotation software, the only UI to aid users was a billboard of the Touch Controllers with a map of each button.

## COMPARISON TO A 3D DIGITIZER ARM

The closer-to-real-world experience in the virtual environment, as provided by our prototype, is comparable to annotating the physical skull with a digitizer arm. When using a digitizer, a landmark is placed by

touching the point on the surface of the skull with the digitizer tip, which is attached to a mechanical arm, and concurrently pressing a foot pedal to automatically import landmark coordinates into a computer. There are notable differences to annotation in VR. With the 3D digitizer tip, an operator touches the skull physically, whereas an operator cannot feel the surface in VR. During physical annotation, an operator cannot move the specimen as this would confound the landmark coordinates, whereas in VR, an operator may freely manipulate the 3D model to find a good annotation angle. Any digital annotation method has the benefit that we can annotate landmarks, which could not be annotated using a physical tool without changing the position of the specimen, simply because the tool cannot physically reach the annotation point.

## REFERENCES

Dominic, J. and Robb, A. (2020). Exploring effects of screen-fixed and world-fixed annotation on navigation in virtual reality. In *Virtual Reality and 3D User Interfaces (VR 2020)*, pages 607–615. IEEE.
