# Supplementary figures and images for: Using virtual reality for anatomical landmark annotation in geometric morphometrics
Source: PeerJ. 2022 Feb 7;10:e12869. doi: 10.7717/peerj.12869 (PMC8830334; doi:10.7717/peerj.12869)

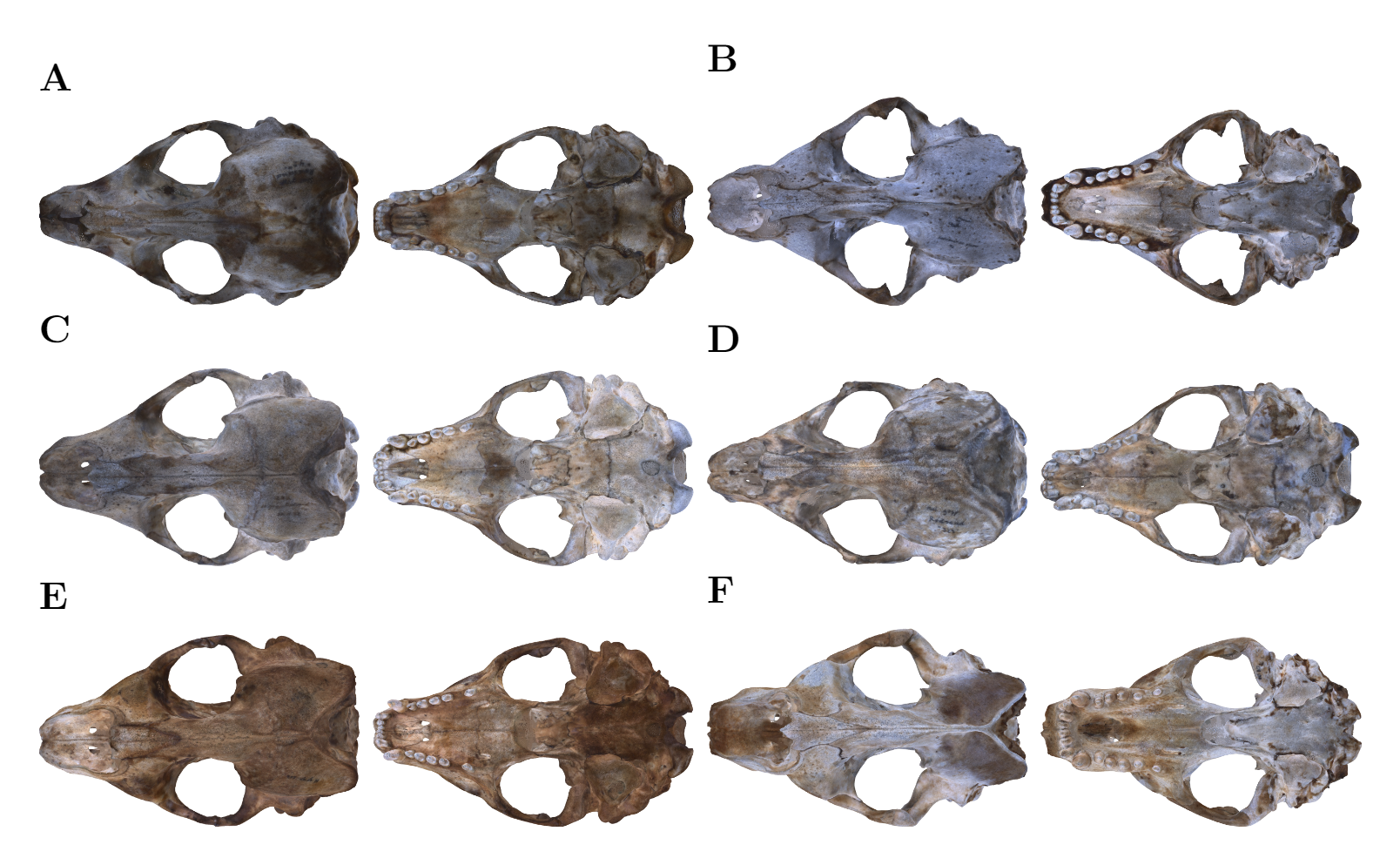

Supplement: Supplemental Information 4 — The six 3D models used for data collection in dorsal (left) and ventral (right) view. (A) Specimen 42.11, (B) Specimen 96, (C) Specimen 232, (D) Specimen 323, (E) Specimen 664, (F) Specimen C7. [file peerj-10-12869-s004.png]

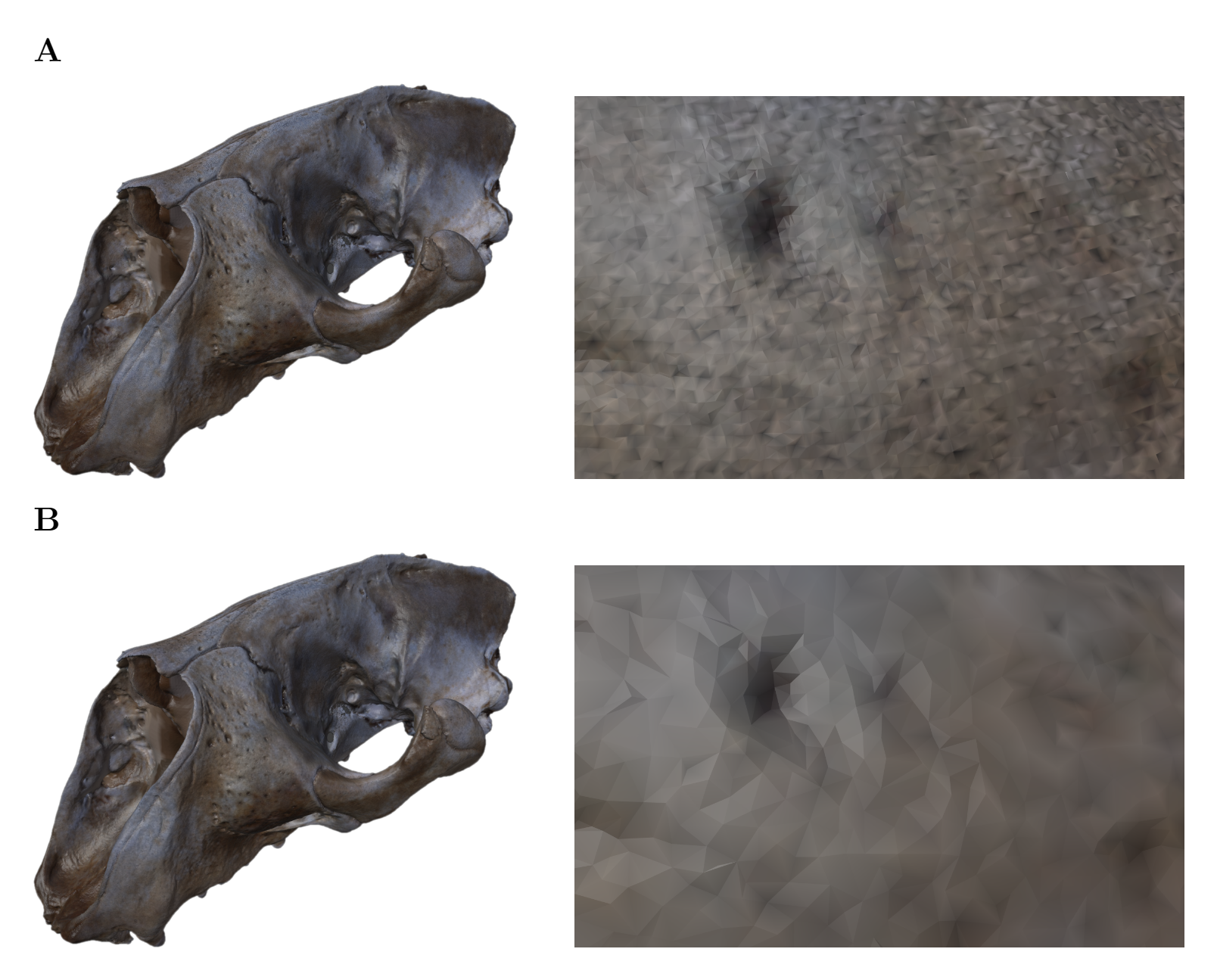

Supplement: Supplemental Information 5 — (A) Original and (B) downsampled 3D model of specimen C7. For each model, we provide a view of the whole model (left), and a close up view (right). [file peerj-10-12869-s005.png]

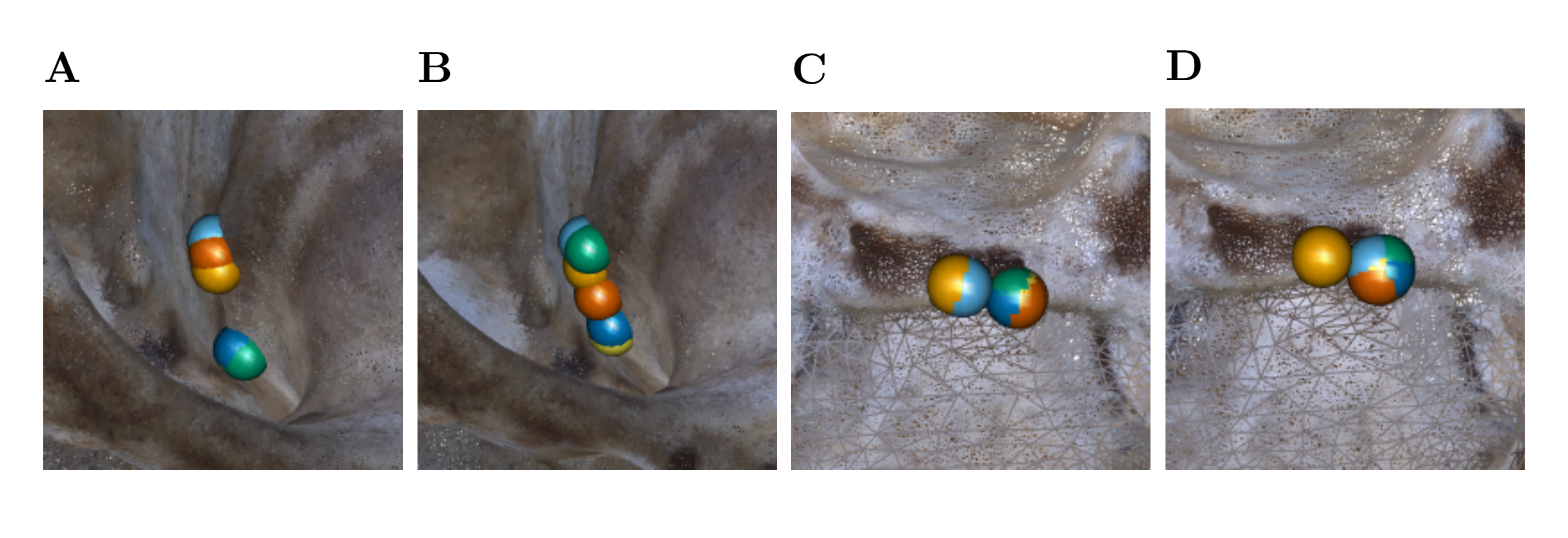

Supplement: Supplemental Information 6 — Six repeated measurements of landmark 28 by operator A on specimen 42.11 using (A) Stratovan Checkpoint and (B) the VR annotation system. Six repeated measurements of landmark 18 by operator C on specimen C7 using (C) Stratovan Checkpoint and (D) the VR annotation system. [file peerj-10-12869-s006.png]
