# Supplementary material for: Using virtual reality for anatomical landmark annotation in geometric morphometrics
Source: PeerJ. 2022 Feb 7;10:e12869. doi: 10.7717/peerj.12869 (PMC8830334; doi:10.7717/peerj.12869)
Supplement: Supplemental Information 7 — The operators were instructed to go through this table column-wise, starting to place landmarks on specimen 323 using virtual reality (top left cell), and ending on specimen C7 using Stratovan Checkpoint (bottom right cell). [file peerj-10-12869-s007.pdf]

Table S1. Pre-defined, randomized order of landmark data collection. The operators were instructed to go through this table column-wise, starting to place landmarks on specimen 323 using virtual reality (top left cell), and ending on specimen C7 using Stratovan Checkpoint (bottom right cell).

System \ Replica	1	2	3	4	5	6
Virtual reality	323	96	223	323	96	223
Virtual reality	223	223	96	96	323	323
Stratovan Checkpoint	96	323	323	223	223	96
Stratovan Checkpoint	42.11	C7	664	42.11	C7	664
Virtual reality	664	664	C7	C7	42.11	42.11
Virtual reality	C7	42.11	42.11	664	664	C7
Stratovan Checkpoint	323	96	223	323	96	223
Stratovan Checkpoint	223	223	96	96	323	323
Virtual reality	96	323	323	223	223	96
Virtual reality	42.11	C7	664	42.11	C7	664
Stratovan Checkpoint	664	664	C7	C7	42.11	42.11
Stratovan Checkpoint	C7	42.11	42.11	664	664	C7
