# Supplementary material for: Using virtual reality for anatomical landmark annotation in geometric morphometrics
Source: PeerJ. 2022 Feb 7;10:e12869. doi: 10.7717/peerj.12869 (PMC8830334; doi:10.7717/peerj.12869)
Supplement: Supplemental Information 8 — There were two outliers, one placed at landmark 11, and 28, respectively. Dependent variable is log-transformed Euclidean distance between single landmark measurements and landmark means. We applied the following crossed structure: System × Operator × Specimen. Residuals reflect landmark replica, and have 240 degrees of freedom. [file peerj-10-12869-s008.pdf]

Table S2. ANOVA, separately for each landmark, without the exclusion of outliers of type 2). There were two outliers, one placed at landmark 11, and 28, respectively. Dependent variable is log-transformed Euclidean distance between single landmark measurements and landmark means. We applied the following crossed structure: System × Operator × Specimen. Residuals reflect landmark replica, and have 240 degrees of freedom.

Variables	Df	F	Pr(>F)	F	Pr(>F)	F	Pr(>F)
		LM 2		LM 3		LM 11	
System	1	2.32	0.129	0.54	0.461	8.65	0.004
Operator	3	21.15	0.000	76.02	0.000	37.46	0.000
Specimen	5	2.36	0.041	42.54	0.000	32.99	0.000
System:Operator	3	0.89	0.445	1.34	0.261	3.49	0.016
System:Specimen	5	2.16	0.059	0.43	0.825	2.60	0.026
Operator:Specimen	15	1.73	0.047	4.20	0.000	7.65	0.000
System:Operator:Specimen	15	0.82	0.657	2.45	0.002	2.85	0.000
		LM 18		LM 24		LM 28	
System	1	1.73	0.190	0.98	0.324	0.13	0.722
Operator	3	31.91	0.000	51.97	0.000	17.49	0.000
Specimen	5	21.60	0.000	12.32	0.000	174.03	0.000
System:Operator	3	0.42	0.741	2.01	0.113	7.73	0.000
System:Specimen	5	0.91	0.476	4.24	0.001	1.93	0.091
Operator:Specimen	15	2.64	0.001	6.18	0.000	8.02	0.000
System:Operator:Specimen	15	1.59	0.077	3.42	0.000	1.52	0.100
