# Supplementary material for: Using virtual reality for anatomical landmark annotation in geometric morphometrics
Source: PeerJ. 2022 Feb 7;10:e12869. doi: 10.7717/peerj.12869 (PMC8830334; doi:10.7717/peerj.12869)
Supplement: Supplemental Information 9 — We applied the following crossed structure: System x Operator x Specimen. Residuals reflect landmark replica. The R-squared values (Rsq) give estimates of the relative contribution of each factor to total shape variation. [file peerj-10-12869-s009.pdf]

Table S3. Procrustes ANOVA on shape, without the exclusion of outliers of type 2). We applied the following crossed structure: System × Operator × Specimen. Residuals reflect landmark replica. The R-squared values (Rsq) give estimates of the relative contribution of each factor to total shape variation.

Variables	Df	MS	Rsq	F	Pr(>F)
System	1	0.00033	0.0003	1.266	0.229
Operator	3	0.00542	0.0160	20.992	0.001
Specimen	5	0.18340	0.8997	710.075	0.001
System:Operator	3	0.00039	0.0012	1.515	0.050
System:Specimen	5	0.00031	0.0015	1.203	0.159
Operator:Specimen	15	0.00113	0.0166	4.364	0.001
System:Operator:Specimen	15	0.00027	0.0040	1.040	0.364
Residuals	240	0.00026	0.0608		
